# Muscle redundancy is greatly reduced by the spatiotemporal nature of neuromuscular control

**DOI:** 10.3389/fresc.2023.1248269

**Published:** 2023-11-08

**Authors:** Brian A. Cohn, Francisco J. Valero-Cuevas

**Affiliations:** ^^1^^ Department of Computer Science, University of Southern California, Los Angeles, CA, United States; ^^2^^ Department of Biomedical Engineering, Division of Biokinesiology and Physical Therapy, University of Southern California, Los Angeles, CA, United States

**Keywords:** motor control, synergies, rehabilitation, neuromuscular activation, manipulation

## Abstract

Animals must control numerous muscles to produce forces and movements with their limbs. Current theories of motor optimization and synergistic control are predicated on the assumption that there are multiple highly diverse feasible activations for any motor task (“muscle redundancy”). Here, we demonstrate that the dimensionality of the neuromuscular control problem is greatly reduced when adding the temporal constraints inherent to any sequence of motor commands: the physiological time constants for muscle activation-contraction dynamics. We used a seven-muscle model of a human finger to fully characterize the seven-dimensional polytope of all possible motor commands that can produce fingertip force vector in any direction in 3D, in alignment with the core models of Feasibility Theory. For a given sequence of seven force vectors lasting 300 ms, a novel single-step extended linear program finds the 49-dimensional polytope of all possible motor commands that can produce the sequence of forces. We find that muscle redundancy is severely reduced when the temporal limits on muscle activation-contraction dynamics are added. For example, allowing a generous ±12% change in muscle activation within 50 ms allows visiting only ∼7% of the feasible activation space in the next time step. By considering that every motor command conditions future commands, we find that the motor-control landscape is much more highly structured and spatially constrained than previously recognized. We discuss how this challenges traditional computational and conceptual theories of motor control and neurorehabilitation for which muscle redundancy is a foundational assumption.

## Introduction

Controlling the muscles of a limb is a task “cursed” by dimensionality, as it is a learning and control problem that requires the nervous system to identify and implement a specific muscle coordination pattern from an infinite set of possible options. Our objective in this work is to add more reality (constraints) to the models to uncover the way muscles “must” coordinate, given a task. This so-called *muscle redundancy* problem has been considered the central problem of motor control in computational neuroscience for the past half century (1).

There are three main conceptual approaches to this problem that attribute the nervous system the ability to mitigate muscle redundancy by (i) *a priori* reducing the dimensionality of the problem to a handful of basis functions or “synergies” [e.g., (2–4)], (ii) defining cost functions to follow a gradient to find unique muscle coordination patterns [e.g., (5–7)], or (iii) using experience-based sampling to find useful coordination patterns [e.g., Bayesian priors (8), trial-and-error (9), or habitual (10)]. Feasibility Theory (11, 12) contextualizes these alternative theories of neuromuscular control by formally describing the high-dimensional set of all neuromechanically feasible coordination patterns. This is the landscape upon which all learning and adaptation must take place at any point in time.

How a neural controller explores and exploits such high-dimensional landscapes is not known. However, from an anthropocentric mathematical perspective (which may not be the way neural systems operate), it is computationally more tractable to use optimization or dimensionality reduction than experience-based sampling, which may take an infeasibly long time in such high-dimensional spaces (13, 14). Moreover, we and others have suggested that there may be non-obvious mechanical constraints that must be considered when selecting coordination patterns such as the integrity of the joint (4, 15), or the instability of the task (16, 17). In the case of rehabilitation, injury and disease likely impose their own mechanical or dynamic constraints.

To find unique time-varying muscle coordination patterns in spite of the muscle redundancy problem, investigators use dynamic optimization and optimal control formulations that enforce tenable (yet arbitrary) convex (generally quadratic) cost function with differential equations that approximate the activation-contraction dynamics of muscle and equations of motion of the limb (6, 7, 18–21). While this approach solves a well-posed mathematical problem, it does not, however, characterize the redundancy problem the nervous system faces: **how** is the *dimensionality and structure* of muscle redundancy affected by the neurophysiological time constants needed to change coordination patterns *over time*?

Synergy analyses are valuable for understanding the tendencies of muscles to collaborate and coordinate with one another, across health and pathologies (22). In monitoring intermuscular coherence between muscles at the 10 Hz (alpha) range, specific postures highlighted more alpha drive than others (23), suggesting that the task has a large effect on the way muscles coordinate with themselves. In the effort to understand the “way” muscles are coordinated, our drive is to clearly and exhaustively characterize the task the neuromuscular system must be solving, so we can uncover the tenets of control. Muscle control and body dynamics must be considered in parallel. This field of work aims to aid in our comprehension of the broader field of comparative physiology and neuroscience, ultimately enhancing our knowledge of the biological underpinnings of movement in diverse organisms and informing robotics, orthopedic, or prosthetic design for improved mobility and quality of life.

**Figure 1:**
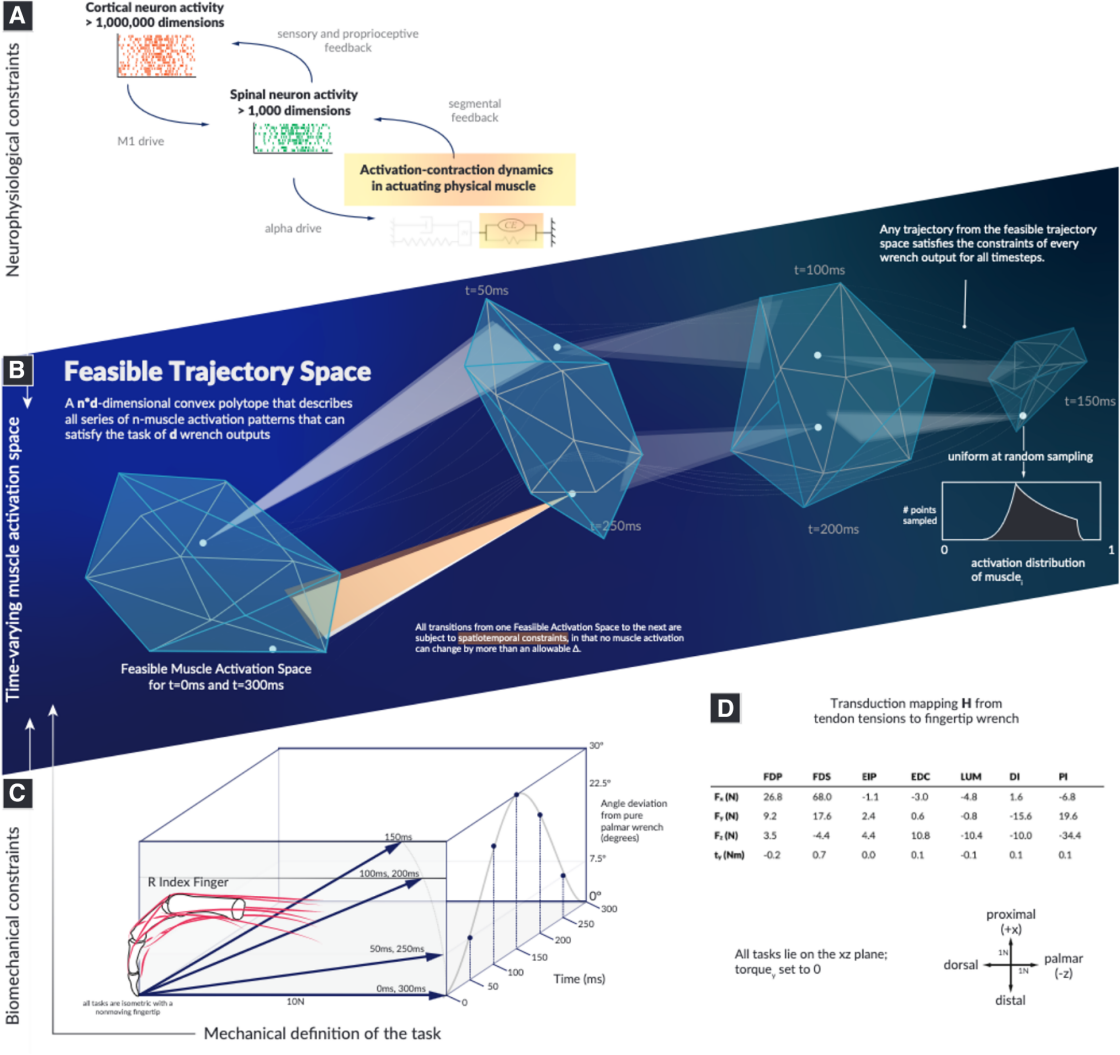
The landscape within which motor learning and performance must occur in time is highly structured and spatially smaller than previously recognized. Our objective is to computationally survey the Feasible Trajectory Space in the context of activation-contraction constraint, to better inform our perspectives of descending neuromuscular control paradigms. We illustrate the types of neurophysiological constraints that affect muscles (**A**), highlight how a feasible activation space is subject to the aforementioned constraints (**B**), select a model system of a human cadaver index finger conducting a force redirection task (**C**), and document the model as well as the axes (**D**). Illustrations in this summary figure are artistic representations.

Here, we describe how muscle activation-contraction dynamics, *a dynamical physiological constraint common to all time-varying limb functions*, affects the dimensionality and structure of muscle redundancy. The dynamics of the limb and task are as diverse as the multitude of behaviors, but the fact that muscles cannot change the force level instantaneously affects the options in the next moment (24, 25). We refer to this behavior as a “spatiotemporal tunnel—the well-structured representation of feasible muscle activations to achieve a series of isometric forces, where the limb must meet the force output across each discretized moment in time (Figure 1). We provide a novel conceptual and computational approach to determine how muscle activation-contraction dynamics limit the feasible changes in muscle activation pattern at a given point during a time-varying force modulation task.

We demonstrate, using the sample task of redirecting a 10N fingertip force over a 30${}^{\circ }$ arc (Figure 1), that we can characterize a well-structured and lower-dimensional “spatiotemporal tunnel” that contains the set of all feasible muscle activations without invoking cost functions or performing *a priori* dimensionality reduction.

## Results

**Figure 2:**
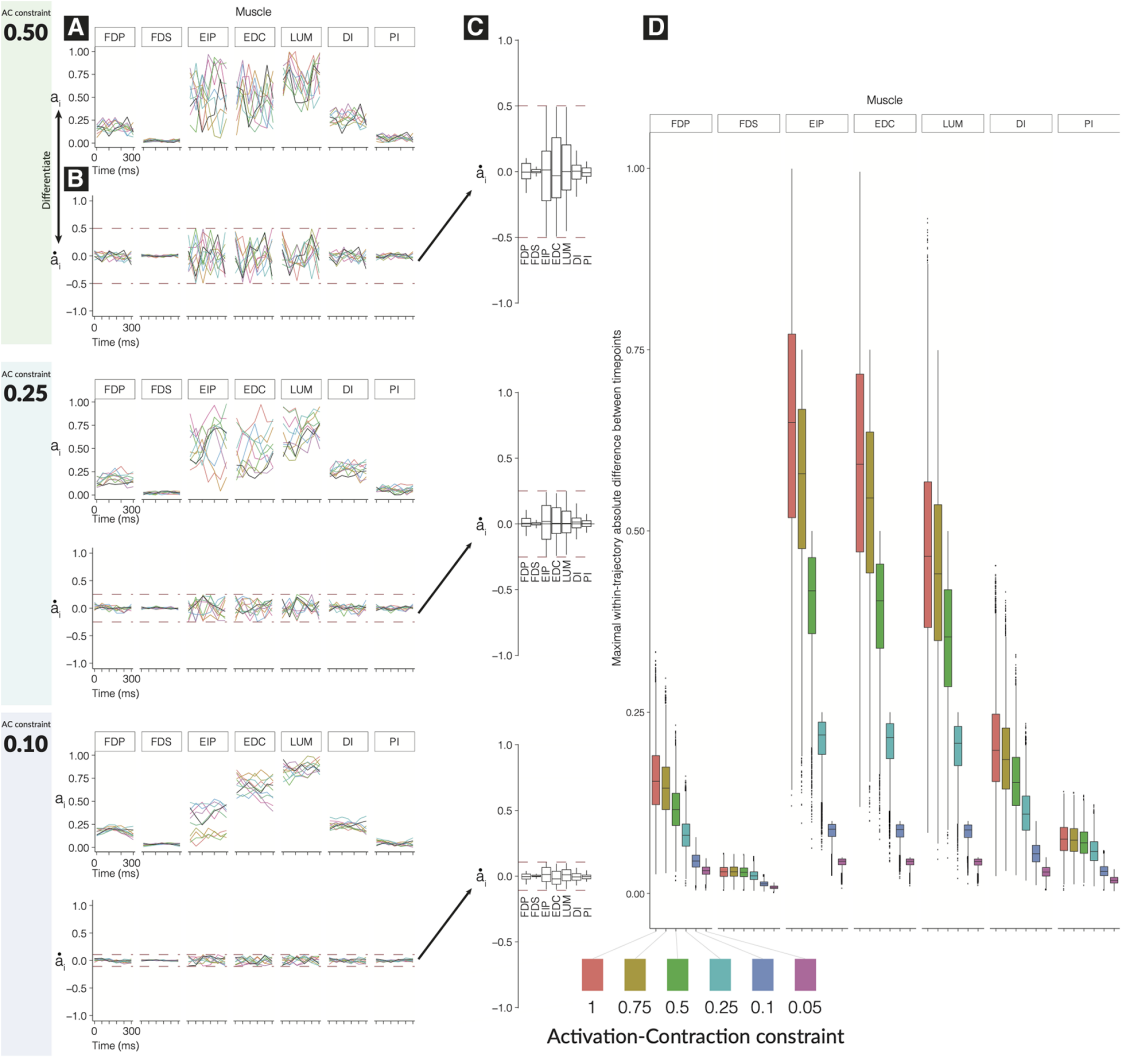
Effect of tightening constraints on the feasible trajectory space. We show 10 sample trajectories (from the 10,000 computed) for each of three levels of activation-contraction constraints. For each activation-contraction level, we show (**A**) sample trajectories, where each color is a different trajectory. (**B**) Those trajectories, differentiated to show how quickly the activations were changing with the upper and lower activation-contraction constraints shown as dotted lines, and (**C**) the full distribution (n=10,000) of the trajectory “activation-contraction speeds,” grouped by muscle. Note that colors on (**C**) do not relate to (**A**) and (**B**). Outliers are not shown on (**C**). In (**D**), we show the effect of differing activation-contraction constraints on the distribution of $\max(|{\dot{a}}_{i}|)$, compared across muscles. When we sample trajectories, we get n-dimensional trajectories, with n=7 muscles. From each of those trajectories, we differentiate them (e.g., ${\dot{a}}_{i}=a_{i+1}^{\mathrm{LUM}}-a_{i}^{\mathrm{LUM}}$), and we show here the distributions of, e.g., ${\dot{a}}^{\mathrm{LUM}}$. These speeds are grouped by the applied activation-contraction constraint. The case with no activation-contraction constraints is a 1.0; a 0.1 means a muscle is spatiotemporally constrained so that it cannot change by more than 10% within 50 ms.

We find that the time history of feasible activations for a time-varying task is highly restricted by the activation-contraction dynamics imposed by muscle physiology (Figure 2).

As the activation-contraction speed limit is reduced, the trajectories become more spatially constrained in the regions of the feasible activation space they can inhabit/exploit (Figure 2D). This allows us to describe the effects of the *activation-contraction constraint* under which muscle coordination happens to be able to produce a force and change its direction on the activation levels across the task, activation-contraction speeds (Figure 2B), signed maximum activation-contraction speeds observed across the larger pool of generated trajectories (n=10,000, Figure 2C), and the max-absolute-value activation-contraction speeds for each muscle, across each activation-contraction constraint (Figure 2D). Some muscle trajectories appear more profoundly affected by more stringent activation-contraction constraints than others, such as extensor indicis propius (EIP), extensor digitorum communis (EDC), and lumbrical (LUM) (Figure 2D). As the maximal activation-contraction speed is reduced, those same muscles will visit/exploit increasingly smaller subspaces of their feasible activation space. This spatiotemporal interaction is best seen in EIP, which has a naturally large range of feasible activation, which are suitably exploited when the activation-contraction constraint is less-constraining, but then shrinks as the constraint becomes more strict. However, changes also spill over to muscles with naturally smaller ranges of feasible activations such as FDP. This muscle has few trajectories with an activation-contraction rate greater than 0.25 to begin with, but becomes limited in range as the activation-contraction speed is reduced (Figure 2D).

A closer look further confirms that muscles that have a narrow range of feasible activations will be least sensitive to changes in activation-contraction constraints. FDP, FDS, and PI are more affected by the reduction of maximal activation-contraction speed—muscles which do not have much room to move are constrained primarily by their involvement in the task, and secondarily by the activation-contraction constraints—the muscles that have greater ranges of activation have non-overlapping central quartiles between the 0.75 and 0.5 activation-contraction constraint (Figure 2D).

**Figure 3:**
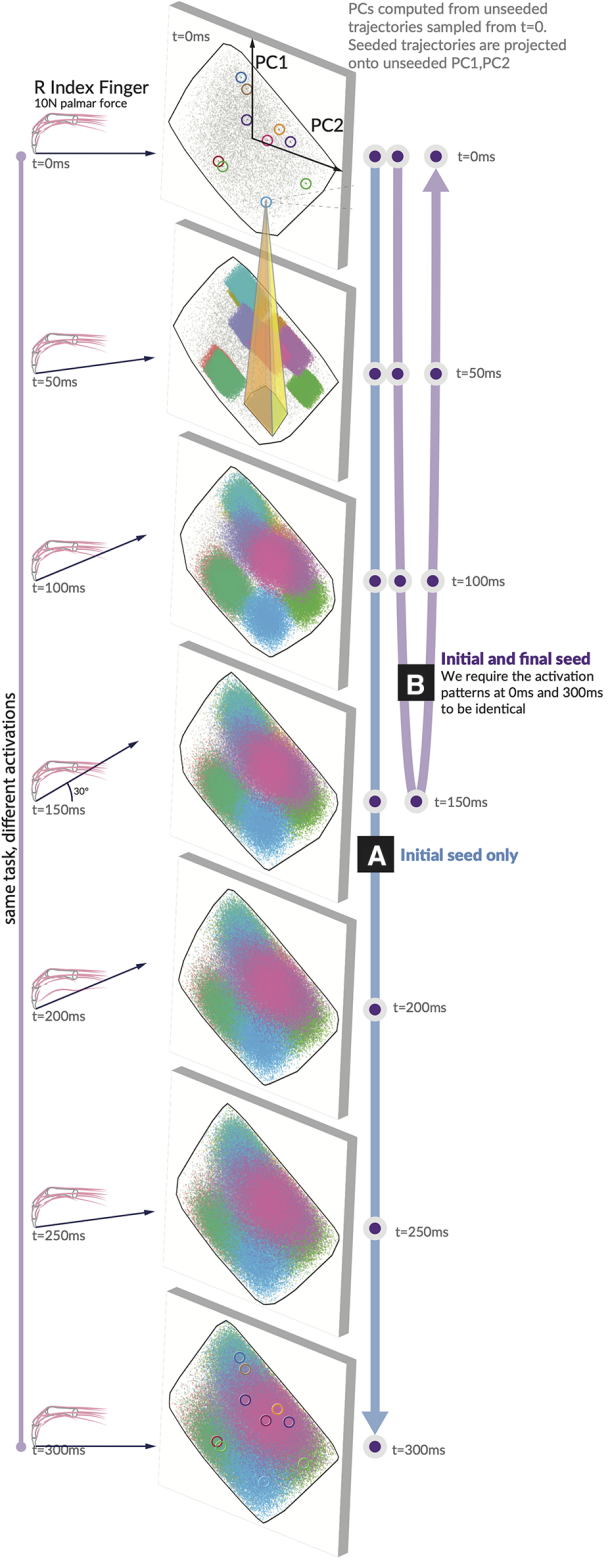
Spatiotemporal tunnels. The ‘seed’ activation you choose in the first moment highly constrains where your muscle activations can evolve across the following six 50 ms time steps. Consider a generous activation-contraction limit of 0.12 per 50 ms time step (in that no muscle can change more than ±12% in tension from slice to slice). We show 10 seeds as they move through the 7 feasible activation spaces, each corresponding to a different force direction. All feasible muscle activations have been projected onto PC1 and PC2 generated from the unseeded distribution for each force direction. Note the color-coded projection of ten sample seeds onto the next time step. A typical example (yellow highlight) shows that only 7% of the next feasible space can be reached when muscle activations are constrained to remain within 12% of its activation from the first seed. We show these spatiotemporal limitations on muscle coordination patterns for (A) the initial value case with only an initial seed activation prescribed, and (B) the clamped case where the initial and final activation match the seed.

Producing a fingertip force and changing its direction require selecting a specific solution and implementing a specific sequence of activation patterns. Our “seeded analysis” reflects the consequences of choosing an initial activation pattern (a “seed”) to subsequent feasible activation patterns. We examine the case where the first activation is fixed to a single option (the unclamped case in Figure 3A) and the “clamped” case, where the first and last activation must match one another (Figure 3B). Given a seed, subsequent feasible activations for each muscle are highly limited in where they can go when unclamped, and when clamped the activation trajectories have (by design) a symmetric expansion and contraction of the activation space. Traditional techniques for visualizing these spaces, including density distributions and parallel coordinates as used in (11), could be misleading on the raw activations when incorporating the concept of time.

Finally, the hypothesis illustrated conceptually in Figure 1 is highly supported by data in Figure 3. We show how, for 10 randomly selected seed points, the activation-contraction constraints shown in Figures 2A,B limit the evolution of muscle activations over time in the force redirection task, and how the blue seeded point has its space limited to 7%. The 7% result is when muscle activations are constrained to remain within 12% of its activation between each time slice (the highest allowable activation change is set to 0.12 per 50 ms).

To create an adequate visualization, we had to find a method to fairly represent, project, and render the 49-dimensional space of trajectories onto a page as a 2D representation.

Given a starting point in t=0, the time-constrained activations form a “tunnel.” A single seed point defines where the activation must move, highly limiting the space of feasible activation patterns that can be used to achieve the rest of the task; a spatiotemporal tunnel exists. Furthermore, when constraining the muscle activation trajectory to have identical starting and finishing activation patterns as in Figure 3B, the muscle redundancy shrinks dramatically.

## Discussion

The temporal constraints imposed by muscle activation-contraction dynamics greatly affect the neuromuscular control landscape upon which all learning, motor control, and evolution must operate. This underappreciated aspect of muscle mechanics has strong implications when navigating the feasible solution space for a task where a null space for control exists. In addition, this approach challenges traditional computational and conceptual theories of motor control and neurorehabilitation for which muscle redundancy is a foundational assumption.

Importantly, the range of valid solutions, i.e., muscle redundancy, does not necessarily decrease in light of activation-contraction constraints, as the feasible activation space is defined by the anatomy of the limb and the operating constraints of the task (12). In addition, the *utility* of a muscle has been described many times as a description of the bounds within which that muscle can be used and can contribute (11, 26, 27, 28). Nevertheless, our work highlights how those bounds on muscle redundancy are too optimistic for time-varying evaluations of spatiotemporal feasibility, even in a very simplistic force redirection task. That is, just because a solution in the feasible activation space is valid, it does not mean that it is reachable at any point in time. Rather, the possibility of implementing a new muscle coordination pattern in finite time is conditioned on the current muscle coordination pattern.

As discussed in detail in (11, 12, 29), the concept of muscle redundancy has been interpreted to mean that the nervous system is confronted with the computational need to solve an underdetermined problem that has infinite solutions. There are, however, constraints other than those explicitly imposed by the force or movement production task that conspire to reduce the dimensionality of the feasible activation space. For example, muscle coordination must consider anatomical constraints to stabilize joints (4, 30) or regulate limb impedance (31)—which additionally reduce the feasible activation set. We now add the critical aspect of *temporal constraints* imposed by muscle activation-contraction dynamics, further showing that the “problem” of motor control is not as underdetermined as commonly proposed. Importantly, “reducing the dimensionality” of the feasible activation is synonymous with meeting an additional constraint (12). This additional spatiotemporal structure in time-varying muscle activation patterns then further complicates the disambiguation of the so-called *descriptive synergies* that arise naturally from the structure imposed by the constraints of the task, from *prescriptive* ones that are proposed to be explicitly regulated by the nervous system (12, 32–34).

Moving beyond the traditional view of muscle redundancy opens up exciting alternative perspectives to understand function, disability, and rehabilitation. It is no longer necessary to continue to assume that optimization or dimensionality reduction of motor signals is the only or primary *modus operandi* of the nervous system (11, 29, 35). For one, our work highlights the important role the properties of muscle play in the co-evolution and co-adaptation of brain and body (36).

More broadly, our results point to the long-underappreciated hierarchical distributed architecture of the nervous system (37–39): the time history of muscle activations arises from the collaboration of slower cortical “higher-level” circuits with faster brainstem and spinal “lower-level” circuits to manage perturbations and other time-critical interactions with the ground and body mechanics. Thus, for example, stroke rehabilitation need not only focus only on disruptions of the corticospinal tracts as they coordinate “redundant musculature.” Rather, we can focus on potentially more clinically impactful approaches for understanding the interactions across the neuroaxis to mitigate the known dysregulation of brainstem and spinal circuitry in stroke (40–42) and the accompanying reduction in rate of force production (43) responsible for deficits in the time-sensitive coordination of muscles.

At a practical clinical level, these findings improve our understanding of the impact of changes in the rate of muscle force production on neuromuscular control, its deficits in clinical conditions, and their rehabilitation. The rate of muscle force production is determined by the recruitment and rate coding of the motoneuron pool combined with activation-contraction dynamics. The net rate of force production can, for a variate of reasons, be enhanced with training (44) or reduced by pain (45), joint injury (46), Parkinson’s disease (47), and stroke (43)—among other conditions. An impact on the feasible activation space can be readily modeled by reducing the strength or speed of a given muscle with the same approach described herein. Therefore, our results directly suggest that the ability to navigate the feasible activation space can be positively or negatively affected by those muscle-level changes from training and clinical conditions, respectively.

This expanded perspective aligns more closely with the complexities of the co-evolution of neural, muscular, and anatomical structures for effective control of real-world motor tasks with realistic muscle activation-contraction properties. In addition, it offers a more comprehensive understanding of how the nervous system collaborates with muscle activation-contraction properties to efficiently control function. Navigating these spatiotemporal landscapes, and how those landscapes change, is the physics upon which animal brains and bodies co-evolved. Our work is thus conducive to cross-species comparisons of “spatiotemporal tunneling” in the context of evolutionary biology like, for example, when comparing the index finger manipulability between humans and bonobos (48). Several cadaveric, computational, and *in vivo* studies then allow a wide variety of future comparisons to support ongoing research into the control of numerous muscles (49, 50), and how it is further constrained by mechanics and time (17).

Prior work in (11) directly shows the impact of reducing the strength of one muscle and can show the relative loss of volume for the resulting feasible activation space—the same analysis could be readily used with the spaces with additional spatiotemporal constraints. For instance, consider a scenario where the strength of a few muscles is reduced by 50%, possibly due to an acute injury. By utilizing models developed in this research, we can visually depict how the remaining muscles might need to adjust to accommodate this altered motor capability while still achieving the same task performance. This can offer valuable insights to clinicians and researchers, helping them better comprehend why even a minor injury can result in a significantly different movement pattern. While the change in one muscle may seem small, it can render certain areas within the range of feasible muscle activations inaccessible. Consequently, the patient will need to find a new solution or opt for a different output force, such as adopting a new walking style.

We use an index fingertip as our “model organism,” and while fewer muscles are involved than for other limbs, there is a path and some precedent for applying these techniques to higher-dimensional models, e.g., the entire posterior chain of a cat (26). Adding activation-contraction dynamics into higher-dimensional systems can help us understand which constraints are most influential in limiting the feasible activation space and help us find which tasks may be more affected.

An important limitation of our work is that additional research is necessary to fully apply our approaches to various scenarios involving pseudo-static, slow, fast, and ballistic movements. We anticipate, however, that incorporating dynamic constraints into our analysis will narrow down the range of feasible muscle activations and provide deeper insights into the actual limitations governing the development and adaptation of motor control.

Ultimately, this paper calls for a measured re-evaluation of existing optimization- and synergy-based motor control theories to better account for how the integrative neuroaxis operates as a hierarchical and distributed system to control the spatiotemporal dynamics of muscle coordination. Producing a more accurate view of the physical system of constraints can aid in our understanding of how motor control has evolved in animals.

## Materials and methods

As in (11, 26, 27), we define the linear transduction of tendon tensions into output endpoint wrench as

(1) $$H\;*\;\bar{x}=\bar{w}.$$

where H (a [4,7] matrix in this paper for four output dimensions and seven input tendon activations) represents the linear activation-to-wrench relationship, such that $H*x={\bar{w}}_{\mathrm{output}}$. We refer to wrench in the mechanical sense, where it represents the output forces and torques produced at the endpoint—in this case, at the fingertip.

Wrenches are four-dimensional as the index finger can produce a torque (i.e., scratching) $\bar{w}=(f_{x},f_{y},f_{z},t_{y})$ (15). We show the output forces, frame-of-reference, and the actual joint torques in Figure 1. As the data for H were collected in the same posture, and as there is strong evidence supporting the linearity of tendon-driven isometric force transduction in fixed postures, we do not need to model the intermediary Jacobian or the Moment-arm matrix (26, 30, 51, 52). We define $x\in [0,1{]}^{7}$, where 1 represents 100% activation.

Note that the term *muscle activation* can take on different meanings depending on the level of the analysis being used. In our case, we use it as shorthand for the total signal needed to produce a given level of neural drive to produce force at each muscle. The reason we do this is that it encompasses the metabolic cost, intensity, and feasible rates of change of both the neural drive and muscle force. As such, it includes the following:

∙ Presynaptic input to a population of α-motoneurones.
∙ The neural command sent by the α-motoneuron to the population of muscle fibers in its motor units (53).
∙ The biochemical processes required for the release and uptake of acetylcholine at the motor end-plate of each muscle fiber (54).
∙ Ca${}^{2+}$ release and uptake by the *sarcoplasmic reticulum* (54).
∙ The cross-bridge cycle at the sarcomere to produce, hold, and change the level of muscle force.

We make the simplification, without loss of generality, to not distinguish between muscle types and consider equal time constants for the increase and decrease of neural drive and muscle force. Many approaches minimize ${\bar{c}}^{T}\bar{x}$, where $\bar{c}$ represents a vector of linear weights to combine with $\bar{x}$ to form a metric of cost, e.g., if $\bar{c}=(1,1,1,1,1,1,1)$, ${\bar{c}}^{T}\bar{x}$ would compute the “sum cost of activation,” or $\bar{c}=(0,0,0,0,0,0,1)$ would compute the “sum of just *palmar interosseus*.” Non-linear objective functions have also been used to better understand weighted $L_{2}$ and $L_{3}$ metabolic cost functions (11). For this paper, rather than minimization or optimization on an arbitrarily defined cost function (a model choice in itself), our approach instead **samples from the nullspace** of $\bar{x}$ uniformly at random (*u.a.r*). We leverage the same computational geometry technique “Hit-And-Run” as in (11, 27), which is originally described in (13).

Synaptic drive applied to motor units create forces, which ultimately generate muscle forces, and accumulate to tendon force. The tendon is compliant and together, the musculotendon is a big dynamic system with many physiological and physical constraints. It is a series elastic element.

### Hit and Run sampling of the feasible activation space

Visualization and analyses of these high-dimensional structures requires unique approaches to highlight different aspects of feasible activation spaces, and there has been some success in using 2D and 3D visualization to decompose neural control of force (11, 27). As the dimensionality of the space increases, the ratio of out-of-polytope to in-polytope volumes within the unit cube expands exponentially, thereby making 2D and 3D approaches computationally intractable with systems with more than two muscles. Like in prior work, we sample the space with the Hit-and-Run algorithm—a Markov chain propagating within the polyhedron that yields a uniform-at-random distribution within the volume of a given convex polytope. This method is agnostic to measures of metabolic or neurologic cost and allows for contextualization of the solutions optimization may select.

### Defining the temporal constraints

One core limitation of our prior work (11) is the single-moment analysis that does not take into account the amount of change the CNS must perform to move from solution to solution, from task to task. Muscles do not act with infinitely fast response times; to respect this, we incorporate an element of temporal constraint in our model by limiting a muscle’s change in activation between ±δ% over a 50-ms interval. Given the observation that deactivation in vertebrate muscle is often slower than activation (53), we set this limit to the faster of the two, forming a conservative bound. We refer to this metric as the activation-contraction constraint, and as we take the absolute value of the deltas, this metric is always set between [0,1]. A constraint value of 0.25 means that in 50 ms, activations can change their output by no more than 25% of their maximal tension.

### Specimen

Our activation-to-wrench model H was sourced from an experiment using cadaver fingers (55), with original data (n=11) from (56). To reveal the effects of activation-contraction constraints on a time-dependent feasible activation space, we leveraged a stochastic Monte Carlo technique to fairly extract activation trajectories—Hit-and-Run (13). In addition to being normalized between an activation of 0 and 1 (muscles cannot go negative as they can only pull), muscles were constrained in their ability to change their output activation from moment to moment. For each moment in time, the endpoint vector had to meet the requirements of its desired output wrench within a series of seven tasks. Formally, we add new constraints in the way the activations can change, which are ultimately classifiable as Lipschitz constraints (57, 58), and we sample u.a.r. (uniformly at random) from the null space on x, given A and b where $x\in [0,1{]}^{n}$. Our Lipschitz constraints (referred to hereafter as “activation-contraction constraints”) serve to link different motor patterns over time to different output wrenches.

(2) $$|x_{i+1}-x_{i}|\leq \delta \;\;x\in [0,1{]}^{7}$$

(3) $$\left(\begin{array}{c} \;f_{x}\\ f_{y}\\ f_{z}\\ \tau_{y} \end{array}\right)=w=Ha=H\left(\begin{array}{c} a_{1}\\ a_{2}\\ a_{3}\\ \cdots \\ a_{7} \end{array}\right),\;\;a\in [0,1{]}^{7}$$

We set the task to a series of seven individual wrenches performed over the course of 300 ms, which starts at a pure $f_{x}$ force (toward palmar), with a 30${}^{\circ }$ rotation toward proximally (rotated about the axis defined by the ulnar direction), and a symmetrical return. The progress is shaped as a single cosine period, with the peak being the fourth index. Wrenches ($w_{t=0}=w_{t=6}$), ($w_{t=1}=w_{t=5}$), ($w_{t=2}=w_{t=4}$) are identical—providing a symmetric set of tasks to stay constant while the activation-contraction constraint demands may change.

### Method for generating unseeded and seeded trajectories

Unseeded trajectories that can originate in any valid solution at t=0 show their evolution across the subsequent polytopes (i.e., solution spaces) subject to the temporal constraints of activation-contraction dynamics of muscles. A seeded trajectory, on the other hand, is pulled from the same constraint matrix, but with an additional constraint: all of the points selected from a seed start at a same seed point (i.e., valid solution at t=0). A seed point can be extracted from the unseeded trajectories. Seeded points can only evolve in time into subregions of the subsequent solution spaces that are reachable given the starting point *and* the temporal constraints of activation-contraction dynamics of muscle. Importantly, unseeded trajectories all meet activation-contraction constraints as well.

### Quantifying the evolution over time of the distribution of solutions for unseeded and seeded trajectories

Here, we detail our method for analyzing and visualizing the effect of selecting a solution seeded in t=0. We began by extracting 100,000 activation trajectories from H (Eq. 3). With 10 of those trajectories, we extracted only the first value and then ran a further sampling paradigm on a modified constraint equation where the first activation pattern (of 7 muscle activations) had to match the seed’s activations at t=0 (unclamped) and another case where the t=0 and t=300 had to match (clamped). As we want to visualize the effect of selecting a seed point, but cannot easily plot a 4D structure embedded in 7D, we applied principal component analysis to each of the seven moments of time across the unseeded distribution. We then projected both the unseeded, and seeded activation trajectories across the first two PCs, highlighting where in the lower-dimensional space those solutions were most probable.

We sampled 100,000 activation trajectories per activation-contraction constraint condition, where the constraints were set from 1 to 0.05.

### Seeded and Unseeded analyses

To address this difficulty in analyzing the distributions of muscle activations, we present the following “unseeded vs seeded” trajectory analysis in Figure 3. We compute the possible trajectories when the first moment is fixed to a seed point and compare those “seeded” trajectories to the “unseeded” trajectories that were not fixed. Unseeded trajectories are still sampled under the same activation-contraction constraint as their seeded counterpart, that all unseeded trajectories meet the activation-contraction constraint, and that all seeds must have a starting point that exists in the unseeded polytope. For the “clamped” case, we require the starting and ending point activations to match one another. To pick good seed points that would generate viable trajectories, we computed 100,000, filtered by those that met an activation-contraction constraint of ∼0.12, and selected 10 at random as our “seeds.” For each seed, we trimmed off the t=50 to t=300 activation values and appended a new constraint to the original constraint matrix, so all sampled trajectories had an additional constraint to match the seed in t=0. Finally, to evaluate the effect of adding this constraint, we examine the t=50 time step between the seeded and unseeded case, tracing the 2D convex hull of the PNG image in pixels [ImageJ (59)]. The inner area (seeded case) of 8,972 px${}^{2}$ divided by the outer area (unseeded case) $126,926\;{\mathrm{px}}^{2}=7\%$.

## Data Availability

The datasets presented in this study can be found in online repositories. The names of the repository/repositories and accession number(s) can be found here: https://github.com/bc/stfeasibility.
