# Supplementary material for: Muscle redundancy is greatly reduced by the spatiotemporal nature of neuromuscular control
Source: Front Rehabil Sci. 2023 Nov 8;4:1248269. doi: 10.3389/fresc.2023.1248269 (PMC10663283; doi:10.3389/fresc.2023.1248269)
Supplement: Supplementary file 1 [file Datasheet1.zip › Data Sheet 1_v1/stfeasibility-Pub/figures/plot_constraints_one_moment.pdf]

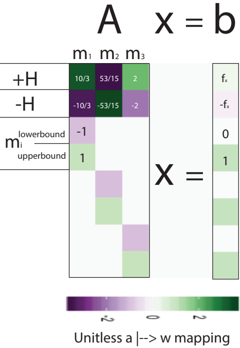

A x = b
m1 m2 m3
+H
-H
lowerbound
upperbound
10/3
53/15
2
-10/3
-53/15
-2
-1
1
fx
-fx
0
1
X =
-2
0
2
Unitless a |--> w mapping

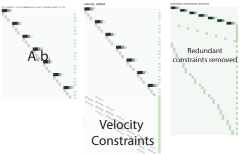
velocity_added
redundant constraints removed
A_i, b_i
Redundant constraints removed
Velocity Constraints
