# Supplementary material for: Muscle redundancy is greatly reduced by the spatiotemporal nature of neuromuscular control
Source: Front Rehabil Sci. 2023 Nov 8;4:1248269. doi: 10.3389/fresc.2023.1248269 (PMC10663283; doi:10.3389/fresc.2023.1248269)
Supplement: Supplementary file 1 [file Datasheet1.zip › Data Sheet 1_v1/stfeasibility-Pub/figures/st_feasibility_boxplots.pdf]

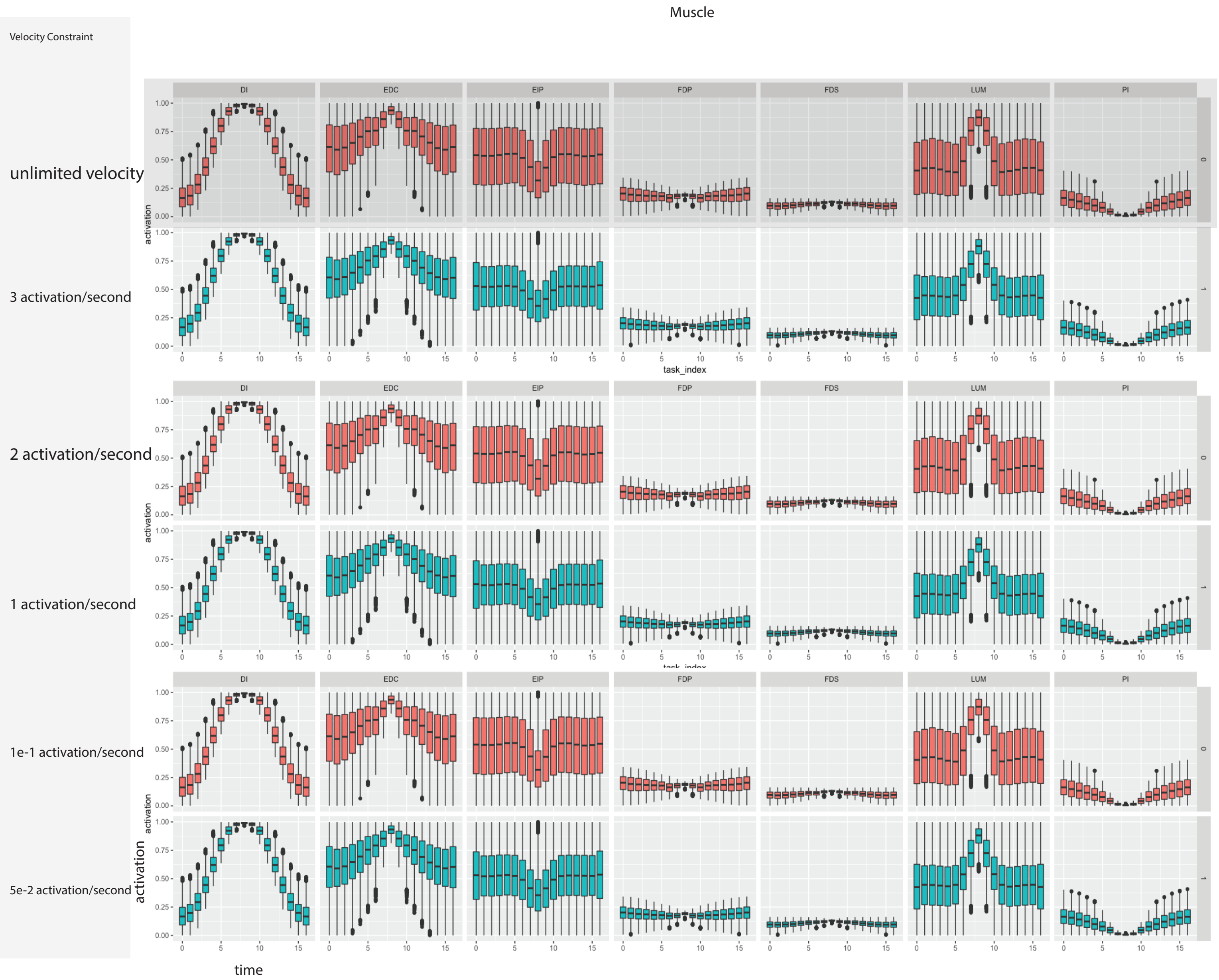

Muscle
Velocity Constraint
unlimited velocity
3 activation/second
2 activation/second
1 activation/second
1e-1 activation/second
5e-2 activation/second
DI
EDC
EIP
FDP
FDS
LUM
PI
activation
task_index
time
