# Supplementary figures and images for: Muscle redundancy is greatly reduced by the spatiotemporal nature of neuromuscular control
Source: Front Rehabil Sci. 2023 Nov 8;4:1248269. doi: 10.3389/fresc.2023.1248269 (PMC10663283; doi:10.3389/fresc.2023.1248269)

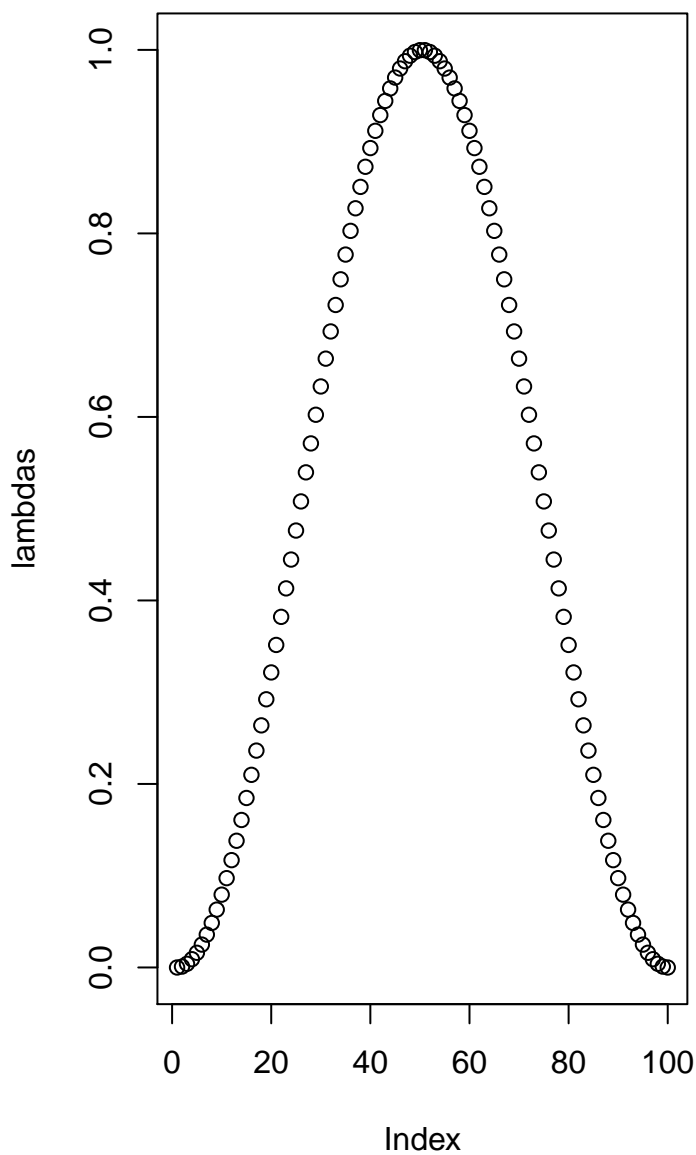

Supplement: Supplementary file 1 [file Datasheet1.zip › Data Sheet 1_v1/stfeasibility-Pub/figures/lamdbas.pdf]

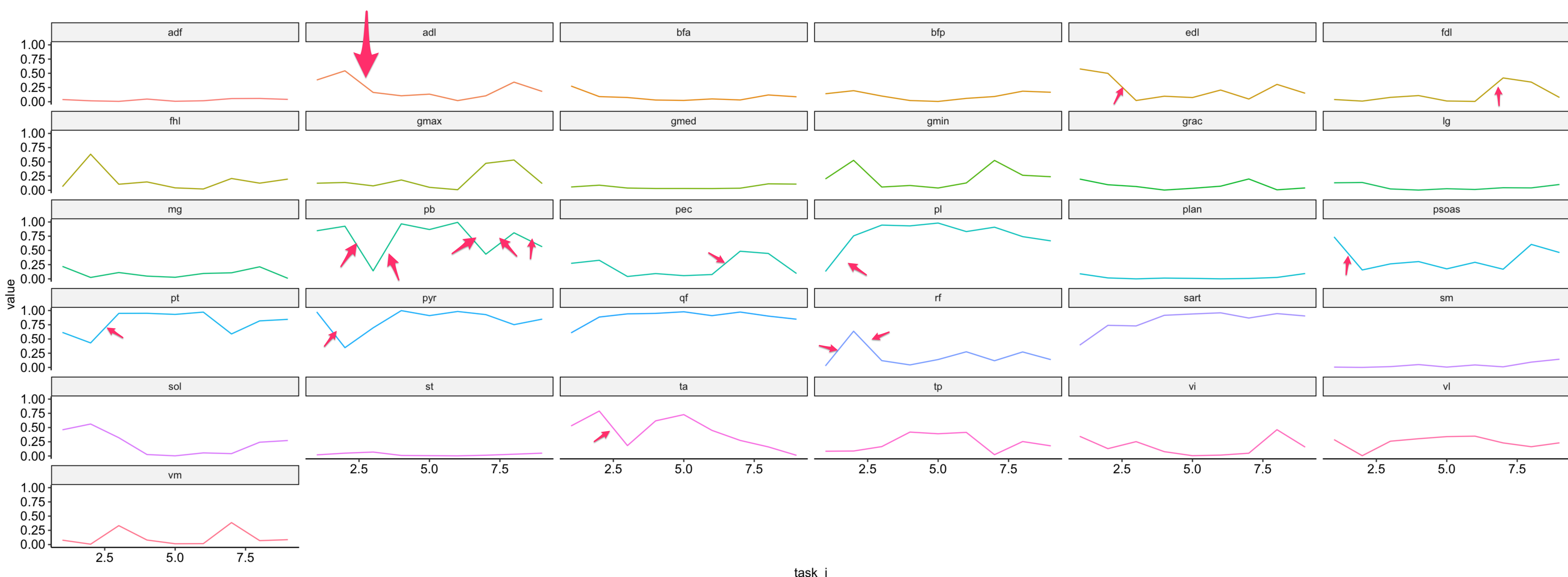

Supplement: Supplementary file 1 [file Datasheet1.zip › Data Sheet 1_v1/stfeasibility-Pub/figures/sample_redirection_task_trajectories_with_labeled_badvelocities.pdf]
